# Achieving opioid-free anaesthesia for laparoscopic cholecystectomy using the external oblique intercostal block—a case series of three cases

**DOI:** 10.1093/jscr/rjaf271

**Published:** 2025-05-04

**Authors:** Chi Ho Chan, Jia Yin Lim, Baldwin Po Man Yeung, Chi Wai Chan

**Affiliations:** Department of Anaesthesiology, Sengkang General Hospital, 110 Sengkang E Wy, Singapore 544886, Singapore; Department of Anaesthesiology, Sengkang General Hospital, 110 Sengkang E Wy, Singapore 544886, Singapore; Department of Surgery, Sengkang General Hospital, 110 Sengkang E Wy, Singapore 544886, Singapore; Department of Anaesthesiology & Operating Theatre Services, Kwong Wah Hospital, 25 Waterloo Rd, Yau Ma Tei, Hong Kong

**Keywords:** anaesthesia, conduction, cholecystectomy, laparoscopic, external oblique intercostal block, nerve block, surgical procedures, operative

## Abstract

Laparoscopic cholecystectomy is associated with significant postoperative pain. The external oblique intercostal (EOI) block is a recently described superficial block that can be performed in the supine position and provides effective pain relief for upper abdominal surgeries. This case series aim to describe the use of the EOI block in providing analgesia following laparoscopic cholecystectomy. We reported three female patients, aged 62 to 71 years, who underwent laparoscopic cholecystectomy under general anaesthesia with an EOI block. None of the patients required opioids for intraoperative analgesia beyond induction. Postoperatively, all three patients reported only mild-to-moderate pain localized to the umbilical port site. Postoperative opioid consumption was minimal. No adverse effects or block-related complications were observed. The EOI block demonstrated promising efficacy in providing analgesia for laparoscopic cholecystectomy. Further studies are warranted to determine the effectiveness of the EOI block for a wider range of laparoscopic upper abdominal surgeries.

## Introduction

Laparoscopic cholecystectomy is associated with significant pain after surgery and is one of the most common causes of readmission after ambulatory laparoscopic cholecystectomy [1]. The external oblique intercostal (EOI) block is a more recently described regional anaesthesia approach to anaesthetize the upper abdomen which is easy to perform and may provide effective analgesia for laparoscopic cholecystectomy. Our case series aims to describe the potential use of EOI block for laparoscopic cholecystectomy. This article adheres to the CAse REport guidelines for case reports, and written consents have been obtained from patients for publication.

## Case reports

### Description of block

**Figure 1:**
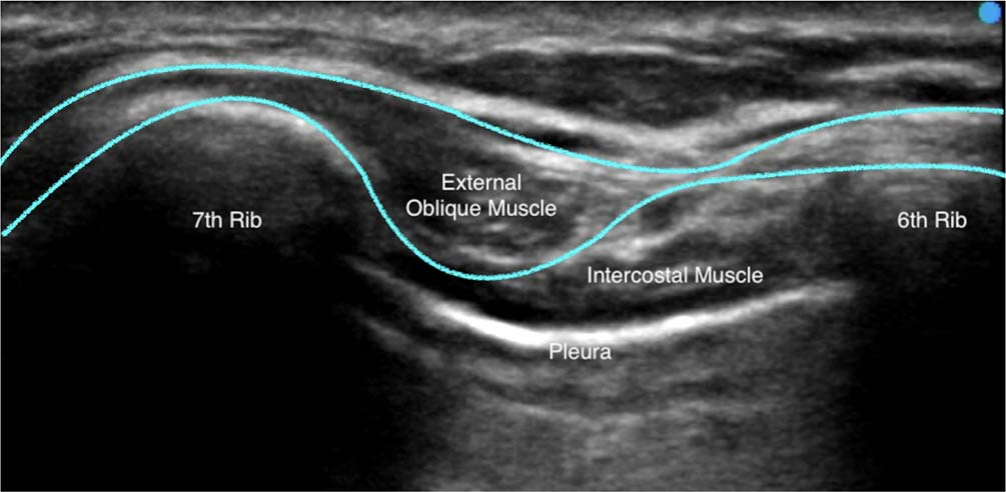
A typical ultrasound image obtained while administering the EOI block. The external oblique muscle, intercostal muscles, sixth rib, seventh rib, and pleura is labelled. The border external oblique muscle border is outlined.

The EOI block was performed with patients in the supine position. The sixth rib was determined using the level of the xiphoid process at the midclavicular line. A Sonosite Edge II ultrasound machine (Fujifilm) was used, and a linear 15–4-MHz ultrasound transducer was positioned in the sagittal oblique plane, with the probe cranial end rotated slightly medially, at the sixth rib level between the anterior axillary and midclavicular lines. The subcutaneous tissue, external oblique muscle, ribs, intercostal muscles, pleura, and lungs were identified from superficial to deep, as seen in Fig. 1. With an in-plane technique, a 22-gauge Sonoplex® STIM single shot nerve block needle (PAJUNK®) was used to inject local anaesthetic between the fascial plane of the external oblique muscle and the external intercostal muscle at the level of the intercostal space between the sixth and seventh rib. A clear separation of the muscle plane between the external oblique muscle and the external intercostal muscle was observed upon the injection of the local anaesthetic, as demonstrated in Video 1.

### Case 1

A 62-year-old Malay female (height 153 cm, weight 69.1 kg, body mass index [BMI] 29.9 kg/m^2^) was admitted electively for laparoscopic cholecystectomy for biliary colic. She had no significant past medical history.

The patient was induced using propofol 180 mg, atracurium 30 mg, and lidocaine 50 mg, while anaesthesia was maintained using sevoflurane. Following induction of general anaesthesia, a right EOI plane block was performed using 30 ml of 0.2% ropivacaine. The laparoscopic surgery was performed with laparoscopic ports inserted in the supraumbilical, epigastric, right-upper-quadrant, and right flank. Intraoperatively, dexmedetomidine with a loading dose of 0.3 μg/kg followed by maintenance infusion of 0.4–0.6 μg/kg/hour was administered. Intravenous (IV) paracetamol 1 g was given at the start of surgery, and no opioids or additional local anaesthetics were used intraoperatively. Surgery was completed uneventfully after 62 minutes.

At the end of surgery, the patient was reviewed in the post-anaesthesia care unit (PACU) by a block proceduralist. She reported mild pain at the umbilical port site with a pain score of 2 out of 10 which did not necessitate further analgesia. In the general ward, she received IV paracetamol 1 g regularly four times a day and requested two doses 50 mg of oral tramadol, once on postoperative day (POD) 0 afternoon and another on POD 1 morning. She was reviewed by the acute pain service (APS) team on POD 1 and reported no pain and no symptoms of nausea. The block receded completely without any observed complications and was subsequently discharged home on POD 1 morning.

### Case 2

A 69-year-old Chinese female (height 148 cm, weight 46.4 kg, BMI 21.2 kg/m^2^) was admitted electively for laparoscopic cholecystectomy due to biliary colic. Her significant past medical history included a right hemithyroidectomy for multinodular goiter.

An EOI plane block was performed pre-induction under mild sedation with 1.5 mg of midazolam. Under ultrasound guidance, EOI block was performed using 30 ml of ropivacaine 0.2%. The patient was induced using propofol 150 mg, atracurium 30 mg, and lidocaine 50 mg, while anaesthesia was maintained using sevoflurane. Intraoperatively, dexmedetomidine was administered with a loading dose of 0.5 μg/kg, followed by a maintenance infusion of 0.85 μg/kg/hour. IV paracetamol 750 mg was given at the start of surgery, and no opioids or additional local anaesthetics were used intraoperatively. Surgery was completed uneventfully after 49 minutes.

At the end of the surgery, the patient was reviewed in the PACU by a block proceduralist. She reported only mild discomfort at the umbilical port site with a pain score of 1 out of 10, which did not necessitate further analgesia. In the general ward, no further analgesia was administered on POD 0, and a single dose of 50 mg oral tramadol was administered on POD 1 morning. She was reviewed by the APS team on POD 1 and reported no pain and no symptoms of nausea. The block receded completely without any observed complications, and she was subsequently discharged home on POD 1 morning.

### Case 3

A 71-year-old Malay female (height 147 cm, weight 82.6 kg, BMI 38.2 kg/m^2^) was admitted for acute cholecystitis and was listed for emergency laparoscopic cholecystectomy. She had a medical history of minor coronary artery disease on aspirin, pacemaker-dependent atrioventricular 2:1 block, hypertension, and hyperlipidaemia. She presented to the emergency department with epigastric pain and fever. Acute cholecystitis was confirmed on computed tomography of the abdomen and pelvis, with no evidence of perforation, abscess formation, or biliary tree obstruction. She was started on ceftriaxone and metronidazole, and IV tramadol was used for analgesia.

She underwent laparoscopic cholecystectomy under general anaesthesia the next day in the emergency operating theatre. Rapid sequence induction with cricoid pressure was performed using propofol 160 mg, fentanyl 50 mcg, rocuronium 50 mg, and lidocaine 50 mg and anaesthesia was maintained using sevoflurane. EOI block was performed on the right side after induction of general anaesthesia using 25 ml of ropivacaine 0.2%; however, the block was performed at the level between the seventh and eighth intercostal space instead of the sixth and seventh intercostal space in attempt to reduce the interference from her large breast. Pacemaker magnet was attached to change the pacemaker into unsynchronous mode prior to start of surgery and removed at the end of surgery. Other drugs given intraoperatively included dexamethasone 8 mg at the beginning of surgery and ondansetron 4 mg and paracetamol 1 g at the end of surgery. No additional opioids or local anaesthetics were used intraoperatively. The laparoscopic surgery was performed with laparoscopic ports inserted in the supraumbilical, epigastric, right-upper-quadrant, and right flank. Gallbladder resection was performed and a 19 F drain was placed to gallbladder fossa. The port site wounds were infiltrated with a total of 10 ml bupivacaine 0.5%. There were minimal hemodynamic changes intraoperatively.

The surgery was completed uneventfully after 74 minutes, and the patient was later reviewed in the PACU by a block proceduralist. She reported a mild sharp pain over the supraumbilical port site with a pain score of 2 out of 10. She claimed to be comfortable and did not need further supplemental analgesia. She was transferred to the high dependency (HD) unit for close monitoring in view of her cardiac history. In the HD, she received IV paracetamol 1 g regularly four times a day but did not request for her on-demand doses of IV tramadol. She was reviewed by the APS team on POD 1 and reported a 2 out of 10 pain score over the supraumbilical port site, and worse pain of 5 out of 10 score on movement during physiotherapy. She reported that she slept well overnight and reported no symptoms of nausea. The block receded completely without any observed complications. She remained stable and was stepped down to general ward on POD 1 and discharged home on POD 2.

## Discussion

The EOI block was first described by Hamilton *et al*. as a thoracic fascial plane block for denervation of the anterolateral part of the upper abdominal wall [2]. The block was further evaluated by Elsharkawy *et al*., which demonstrated that an injection of 30 ml consistently covers the lateral cutaneous nerves at the level of T6–T10 and the anterior cutaneous nerves at the level of T6–T9 ± T10 [3]. This technique has largely supplanted the oblique subcostal transversus abdominis plane (OS-TAP) block for upper abdominal surgery, as recent studies have shown that the OS-TAP block provides effective coverage of only the anterior cutaneous nerves and fails to anaesthetize the to the upper lateral abdomen innervated by the lateral cutaneous nerves [4, 5]. Currently, the EOI block is a popular option for upper abdominal surgery and has been described for open cholecystectomy [6, 7], open liver surgery [8, 9], open kidney donor transplantation surgery [10], and open pancreatectomy and splenectomy [11]. It was also described as a rescue block for severe pain after open distal pancreatectomy [12]. Moreover, its distance from the surgical site allows for preoperative catheter placement to provide intra- and postoperative analgesia.

Yet, the EOI block has not been commonly used in laparoscopic upper abdominal surgeries. This is because EOI block is not expected to provide sympathetic visceral coverage that is provided by neuraxial or paraspinal blocks. The PROSPERO review update for evidence-based management of pain after laparoscopic cholecystectomy published in 2018 showed inconclusive results with OS-TAP block and recommended against regional anaesthesia for routine analgesia [13]. However, emerging evidence suggests that the more recently described EOI block may prompt revision of this recommendation. In 2023, Korkusuz *et al*. conducted a randomized controlled trial comparing bilateral EOI block and standard multimodal analgesia in patients undergoing laparoscopic cholecystectomy and found significant reduction in tramadol consumption and better pain score at rest and during movement [14]. In addition, in 2024, Mo *et al*. performed a non-inferior double-blinded placebo-controlled trial comparing bilateral EOI block with rectus sheath block and bilateral OS-TAP block with rectus sheath block in patients undergoing laparoscopic cholecystectomy and found significant postoperative 24-hour reduction in sufentanil consumption and better pain scores [15]. In both of these trials, intraoperative opioids and postoperative opioids were used. In our case series, we demonstrated that the EOI block has a remarkable efficacy in providing analgesia for laparoscopic cholecystectomy with the ability to achieve opioid-free anaesthesia intraoperatively. In all our three cases, fentanyl was only used in one of the cases for endotracheal intubation which could have been avoided using other techniques, and none of our patients required any additional short or long-acting opioids in the perioperative period. Lim and Chan published a case report on the use of the EOI block for open total pancreatectomy and splenectomy, hypothesizing that the EOI block may also provide visceral analgesia [11]. The findings from our case series of three patients appear to support this hypothesis.

From our experience with the EOI block, a key advantage is its superficial nature, ease of rapid identification, and quick and easy to perform. Even in our patient with a BMI of 38.2 kg/m^2^, the EOI plane was only 2–3 cm deep from the skin, allowing easy visualization of the surrounding anatomy and the needle during the procedure. Nevertheless, we did face some challenges in performing the block in female patients with large breast tissue, requiring additional personnel to assist with retraction to access the block site or even performing the block at the level between the seventh and eighth intercostal space instead. Nonetheless, the block remained highly effective despite the altered location of injection.

The patients in our cases complained of mild-to-moderate pain over the umbilical port site while recovering in the PACU. Apart from the umbilical port, discomfort at other sites appears well controlled with local anaesthetic infiltration and systemic non-opioid analgesics. This is consistent with the anatomical evaluation by Elsharkawy *et al*. which showed unreliable coverage of the T10 anterior cutaneous nerve [3]. We recommend administration of simple analgesia in addition to umbilical port site local anaesthetic infiltration at the end of surgery after EOI block for optimal analgesia after laparoscopic cholecystectomy.

In conclusion, the EOI block is effective for analgesia in laparoscopic cholecystectomy and should be considered in routine perioperative strategies. Further research may explore its role in other laparoscopic upper abdominal surgeries and compare its efficacy with blocks offering sympathetic visceral coverage, such as the erector spinae plane block or paravertebral blocks. Cadaveric studies may also help clarify its anatomical basis for such coverage.

## Supplementary Material

Video_1_rjaf271 Video demonstrating administration of the external oblique intercostal (EOI) block.
